# Considerations for Assessing the Appropriateness of High-Cost Pediatric Care in Low-Income Regions

**DOI:** 10.3389/fped.2018.00068

**Published:** 2018-03-27

**Authors:** Andrew C. Argent

**Affiliations:** Paediatric Critical Care, Paediatrics and Child Health, University of Cape Town, Red Cross War Memorial Children’s Hospital, Cape Town, South Africa

**Keywords:** ethics, low- and middle-income countries, high cost, intensive care, children

## Abstract

It may be difficult to predict the consequences of provision of high-cost pediatric care (HCC) in low- and middle-income countries (LMICs), and these consequences may be different to those experienced in high-income countries. An evaluation of the implications of HCC in LMICs must incorporate considerations of the specific context in that country (population age profile, profile of disease, resources available), likely costs of the HCC, likely benefits that can be gained versus the costs that will be incurred. Ideally, the process that is followed in decision making around HCC should be transparent and should involve the communities that will be most affected by those decisions. It is essential that the impacts of provision of HCC are carefully monitored so that informed decisions can be made about future provision medical interventions.

Ideally, every child in the world should have access to an intensive care unit with facilities for endotracheal intubation and mechanical ventilation. No ethical justification exists for providing these treatments to children in rich countries while denying them to children in poor countries (Shann, 2011)

## Introduction

While there is no ethical justification for differences in health-care access for children across the world, dealing with the realities of those differences remains profoundly challenging. During 2016, 5.6 million children under the age of 5 years died across the world (this is equivalent to 15,000 under-5 deaths per day), mostly in low- (LICs) and low- and middle-income countries (LMICs). The majority of those deaths could have been prevented using simple and affordable interventions (1). There are huge differences in under-5 mortality between high- and LICs as well as between higher- and lower-income groups within individual countries (2), in both high- (3) and LICs (4). Addressing intra-country differences could make nearly as much difference to child mortality as addressing the differences between countries. There have been substantial improvements in child survival across the world with the implementation of the millennium development goals, and with those gains, the role and importance of critical care in low- and middle-income countries (LMICs) has increased (5). However, the decision to provide high-cost care in low-income regions is a complex issue where the outcomes and consequences of that provision may be unpredictable and substantially different to those experienced in high-income countries.

High-cost medical care (HCC) incorporates a wide range of medical interventions, and the “value” that is associated with the provision of HCC must take into account many factors including costs, and benefits and the priorities of the people living in those areas. The “high cost” has to be viewed in relation to the available resources and in relation to the value delivered by the care (it is possible to have both high cost and high value, and high cost and low value). Some have argued that there are no “universal” bioethics (6, 7), but perhaps there may be validity in creating some transparency around the processes that are involved in making decisions as to how resources are allocated and on what basis.

In this review, I will consider the health-care context of LICs, the costs and benefits associated with the provision of HCC (in general, but more specifically directed at pediatric critical care), the way in which different people may be affected by HCC, and consider how the process of decision making around resource allocation could be addressed in LICs. Resources are limited in all regions, but there are substantial differences in resource availability, specific contexts, and particular demands on health-care systems across the world.

## The Context

Decision making around the utilization of resources for HCC is deeply affected by a wide number of issues including the underlying context (particularly in terms of disease profile and morbidity and mortality data), the resources available (both absolute and relative amounts), and the potential benefits associated with the HCC (to the child, the family, the community, and the overall health services). An underlying concern with the process of “lumping” countries and groups of countries or communities into simple categories (such as LICs) is the fact that there are huge differences in context between (and within) countries, even within similar income groupings. It is not only the current reality but the “trajectory” of development in a country that may affect which health-care services can and should be provided.

### The Children/Patients

As shown in Table 1, approximately 60% of the world’s children live in low- and LMICs, with some 275 million children living in low-income regions of the world. In those regions, children making up a very substantial proportion of the population have limited access to health-care services and extremely limited access to high-cost health-care services. Health-care expenditure in these regions averages US$35 per capita per annum, and the under-5 mortality is approximately 70 per 1000 live births. The majority of childhood deaths occur in these countries, and various authors have highlighted the fact that the majority of these deaths could be prevented by the implementation of relatively low-cost (and affordable) interventions.

**Table 1:** Data on population, mortality, and health expenditure by income region (8).

Region by Income	% population 0–14 years (2016)	Total population 0–14 years (2015)	Gross national income (GNI) per capita (US $, 2016)	Health expenditure per capita (US $, 2014)	Under-5 mortality (per 1,000 live births), 2016
Low income	42.67	275 365 283	$612	$35	73.1
Lower-middle income	30.83	923 253 971	$2,079	$90	50.7
Upper-middle income	20.60	528 220 719	$8,210	$506	14.1
High income	16.7	197 950 251	$41,046	$5,266	5.4

*As of July 1, 2016, low-income economies are defined as those with a GNI per capita, calculated using the World Bank Atlas method, of $1,025 or less in 2015; lower-middle-income economies are those with a GNI per capita between $1,026 and $4,035; upper-middle-income economies are those with a GNI per capita between $4,036 and $12,475; high-income economies are those with a GNI per capita of $12,476 or more*.

### The Environment

LICs have many features which make the delivery of health care challenging including limited financial and personnel resources for health care, geographical features which may make transport and access challenging, political instability, limited infrastructure (water, electricity, sanitation, transport services, and transport infrastructure), and limited organizational and administrative infrastructure. The large number of displaced people and refugees also complicates decision making for care delivery.

The disease profile in LICs may be substantially different to that in HICs (9–11) and is also changing. In general, there is more trauma (including burns), and there are more infections (including more non-bacterial infections such as dengue, malaria, trypanosomiasis, etc.), and more infections with antibiotic-resistant organisms. Particular concerns relate to infections such as drug-resistant tuberculosis. Data on non-infectious disease are limited, but there is no reason to believe that there is a lower incidence in poorer countries.

As a consequence of multiple factors, patients often present late for therapy (12, 13) with the result that disease processes are often substantially advanced at the time of presentation. A high proportion of deaths occur soon after admission (14). Resource limitations translate into a low number of health-care workers and particularly health-care workers with specialist skills in areas such as pediatrics and pediatric surgery (see Table 2). These workers are deployed in environments that may be overcrowded and poorly maintained (and thus difficult to keep clean), uncomfortable (including heat and humidity or even cold), with very limited equipment and medication. The health-care facilities are often situated in contexts where there may be limited and unreliable sources of clean water, sanitation, and power (particularly electricity and lighting). In a recent review of hospitals in sub-Saharan Africa, the percentage of hospitals with dependable running water and electricity ranged from 22 to 46%, and in countries analyzed, only 19–50% of hospitals had the ability to provide 24-h emergency care (15).

**Table 2:** Resources for health care by income region (8).

Region	Nurses and midwives (per 1,000 population), 2012	Physicians per 1,000 population (2012)	Specialist surgical workforce per 1,000 population (2014)	Out of pocket expenditure (% of total expenditure on health)
Low income	0.69	0.20	0.88	37.19
Lower-middle income	1.77	0.73	10.23	54.87
Upper-middle income	2.80	1.96	39.55	32.39
High income	8.73	2.94	69.28	13.34

Limited and often dysfunctional supply chains contribute to nonavailability of resources that would be taken for granted and assumed in HICs. All of this may be aggravated by political instability and limited personal safety. In the event of natural disasters or outbreaks of infectious disease, these systems may fail spectacularly as has been demonstrated during recent outbreaks of Ebola virus infection in West Africa (16).

All the factors above need to be taken into consideration when contemplating resource allocation for high-cost care.

## Resources for Health Care

In considering the ethics of high-cost care in LICs, it is essential to balance the resources that are available for that care with the outcomes that can be achieved using those resources.

### Financial

**Figure 1:**
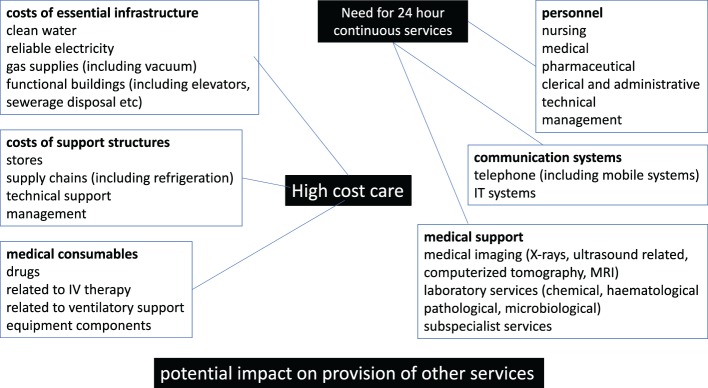
Components of the costs related to high-cost care (HCC) in low- and middle-income countries and low-income countries.

**Figure 2:**
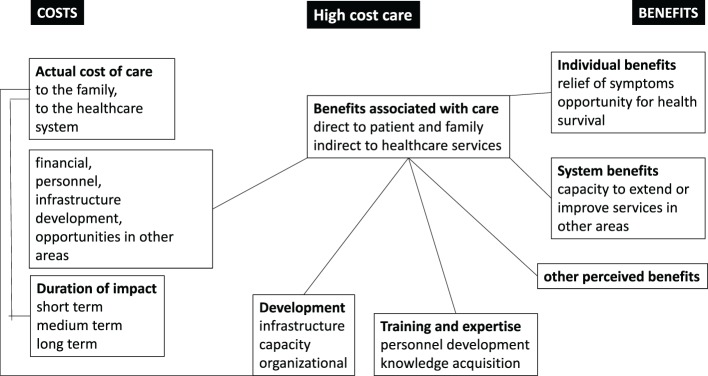
The balances of costs and benefits for high-cost care (HCC) in low- and middle-income countries and low-income countries.

The financial resources available for health care can be considered from a number of perspectives (see Figures 1 and 2). There may be complex interactions between multiple factors.

As shown in Table 1, there is a >10-fold difference in the annual per capita health-care expenditure between LMICs and high middle-income countries and >100-fold difference between LMICs.

In LICs, a significant proportion of the funding available for health care may come from outside the country (see Table 2). That funding may be useful but is usually to be used in ways that are defined by the sources of the funding. Increasingly, funding may also be sourced from research projects that are based on high-income countries. This means that there are external drivers of how that funding can and should be used for health-care provision.

There are different ways of assessing the relative costs of high-cost services. When considering the costs of intensive care in 2009, Baker et al. estimated the costs of a day in intensive care in rich countries to be of the order of $1,000 (17). From a national perspective, that day cost would be approximately 20% of the annual per capita expenditure on health in a rich country but approximately 30 times the annual per capita expenditure on health in an LIC.

The costs of delivering some aspects of critical care in poorer countries may be substantially lower than this, as an example the cost of a day in a private ICU for cancer patients in India was reported as $57, However, in settings where a substantial proportion of health-care costs are covered by out-of-pocket expenditure by families (Table 2), that amount has to be related to the family income. In this setting, $57 was approximately 100× the average per capita household income (18). Thus, the provision of expensive health-care services to individuals in these settings has the potential to devastate the financial structure of a family with severe impact on other family members (including siblings) (19).

Significant as this overall difference is, it may actually mask other differences in costs for HCC. The HCC costs in rich countries reflect the costs of providing that care in the context of systems with well-established and functional infrastructure. In LICs, the infrastructure required (including transport, electricity, water provision and sewerage disposal, medical gas supply systems, technical maintenance support, etc) to support complex medical care may be profoundly deficient. The real cost therefore of providing HCC in “austere conditions” may be substantially higher than currently estimated. This may, however, be offset by the lower salaries that are paid to health-care workers in poorer countries. As another example, it has been argued in HIC that catheter closure of ostium secundum atrial septal defects is substantially cheaper than surgical closure, but in fact this was not the case in Guatemala (20).

Many of the resources required for more expensive therapies (including equipment and medication) are developed and manufactured in high-income countries. Access to these resources may be limited by the direct costs, which are exacerbated by indirect costs such as transportation, import duties and taxes, and adverse financial exchange rates.

In many LMICs, the differentials in access to high-cost health care between rich and poor people may vary substantially. In many countries, a small proportion of the population have access to state-of-the-art medical services while the majority of people within the same country may have limited or virtually nonexistent access to health-care services (21). In South Africa in 2015, approximately 49.8% of total health-care expenditure was from private sources, and only approximately 16% of the population have private health-care insurance (22). This difference in financial resources translates into major differences in access to facilities such as intensive care beds (21, 23).

The widespread corruption in some LICs and LMICs may have a profound effect on the resources actually available for health-care services (24).

Thus, any analysis of the resource implications for HCC in LICs must include a review of all the multiple factors and details in specific environments that may profoundly affect both the absolute and the relative costs of HCC in these countries.

### Personnel

Not only are health-care systems in LICs limited by financial resources but they also have profound challenges as regards the availability of trained and skilled health-care providers. Some years ago, the WHO estimated that the world faced a global shortage of almost 4.3 million doctors, midwives, nurses, and other health-care professionals (25). There are particular shortages in the area of surgery [including pediatric surgery (26, 27)] and anesthesia (28) and rehabilitation personnel (29) in poorer countries. The availability of these health-care workers (particularly to patients who cannot afford private health care) may be profoundly affected by the different remuneration patterns and the fact that many health-care workers have to work in several sectors in order to obtain an adequate income (30).

The standard of care that is (and can be) expected from health-care workers may vary substantially. In most HICs, it would be assumed that staff on out-of-hours duty would be fully awake and present throughout their working time. In many LICs, there is an expectation that afterhours staff will be heavily reduced in numbers relative to the day and that they will be expected to sleep for at least some of the time on duty (after all, many have day jobs that they also have to attend to).

When 24-h services are essential for the provision of HCC (such as pediatric intensive care), then personnel may be both a limiting factor as regards availability, but also as regards costs (four to five people have to be employed for each position that needs to be filled on a 24-h basis).

With substantial differences between financial resources in private and government sectors in LMICs, there are major pressures for health-care workers to move into the more affluent areas either full-time or part-time. This process may substantially decrease the access of less affluent members of society to health-care services that are high cost and often require high levels of training and expertise.

Decisions about HCC in LMIC and LICs may also have an effect on medical emigration. As an example, individuals with training in areas such as surgery or cardiac surgery are likely to emigrate or to move into the private sector if they are consistently unable to operate because of the lack of availability of theater time or PICU beds.

### Structures

Many of the structures in terms of policies, programs, and training infrastructure that are required to support intensive care therapies are simply not present in LICs, as shown in a study completed in Tanzania (31). Thus, time and effort will have to be expended to put all those structures in place before particular services can be provided.

## Outcomes of High-Cost Services

The outcomes of high-cost services are not always simple to establish and may relate to the underlying conditions (see Table 3): the specific interventions undertaken, and the expertise and experience of the teams undertaking those interventions. Some high-cost interventions may be associated with excellent outcomes and minimal long-term costs. At the other extreme, high-cost interventions may be associated with poor outcomes and high long-term costs.

**Table 3:** High-cost interventions, cost, and outcomes.

Example	Short term	Medium to long term	Outcomes
Intensive care for croup	High cost for intensive care (usually only a few days)—but extreme variability in the costs incurred (32)	No expected ongoing costs	Normal life expectancy, small proportion will have recurrent croup

Intensive care for Guillan–Barré syndrome	High cost for intensive care (may require months of ventilation)	May need high input for rehabilitation	Expected to return to normal quality of life with normal activities (may have residual weakness). Some patients have recurrent disease (33–35)

Intensive care for pneumonia or infection	High cost for intensive care (usually a few days but may be longer)	If not underlying disease, minimal long-term costs	Depending on context, may have substantial mortality in hospital and in the 6 months following hospital discharge (36) [particularly if concurrent malnutrition (37)]. However, high chance of normal long-term outcome

Intensive care to enable major surgery	High cost for surgery and intensive care (usually only a few days)	Depending on underlying problems, may be a significant range of long-term costs	The outcomes of a major surgery can be very variable depending on a variety of factors including surgical training and surgical caseload

Surgery for congenital heart disease	High cost for surgery and intensive care	If curative surgery, then minimal long-term costs. May have substantial costs for ongoing care (38) in complex conditions	If successful, excellent outcomes with essentially normal life expectancy and quality of life

Surgery for rheumatic heart disease	High cost for surgery and intensive care	Relatively high costs for ongoing follow-up and medication	Limited long-term survival and high morbidity (39)

Surgery and Intensive care for trauma including burn injuries	Relatively high cost for surgery and intensive care	Depending on the site and extent of the injuries, the long-term costs could be minimal or very substantial	The outcomes may be variable. In the absence of long-term rehabilitation, and in the absence of facilities such as access to cadaver skin or expensive skin replacements, the outcomes of major burns may be extremely poor

There is evidence that relatively low-cost interventions in the care of critically ill children such as the provision of antibiotics at community level to neonates (40), the provision of oxygen therapy to children with pneumonia (41), the provision of high-flow humidified nasal oxygen or nasal CPAP (42, 43), and the improvement of organization of emergency services or of pediatric services within a hospital (44) may be associated with substantial reductions in mortality without significant added expense. Recent neonatal data suggested that focused implementation of nasal CPAP in Nicaragua could provide improved outcomes while reducing invasive mechanical ventilation (45).

Mechanical ventilation has been described in many reports from LICs as being associated with relatively high mortality (46, 47). Thus, in many situations, HCC such as ventilation may not improve overall outcomes as much as simpler and less-resource intensive care modalities.

Where intensive care services have been implemented in LICs, there is some evidence that the strict application of quality control programs can substantially improve the outcomes of pediatric intensive care (48, 49).

## Ethical Principles to be Applied

Having reviewed the context, the resources available, and the potential impact of HCC such as pediatric intensive care, it is important to consider ethical principles that could be considered in making decisions about the provision of HCC (see Table 4) as well as the characteristics of the processes used in decision making (Table 5). Turner et al. (14) have highlighted the principles of global justice, resource allocation, and local cultural preferences. Clearly, there is no ethical justification for the global differences in access of children to HCC (50), and there is a huge need to provide advocacy for additional resources from HIC to be allocated to LIMCs, but that will (at best) take time. However, there is a need to review both the basis and the processes used for resource allocation both within and between countries.

**Table 4:** Ethical principles to be applied in decision making around high-cost care (HCC).

Principle	National level	Community level	Individual level
Respect for autonomy	Rights of nations to make decisions regarding the prioritization of health services in that country.	The rights of communities to be involved in the processes that affect what and how medical care will be delivered to them	The rights of individuals and their families to make decisions regarding issues that affect them

Beneficence	The HCC should provide an improvement in the quality of life in that country	The HCC should improve the quality of health and life in the community which is being provided with that service	The care that is offered has to be seen to provide value to the individual child and his/her family

Non-maleficence or “do no harm”	The provision of the particular HCC cannot be seen to endanger the delivery of other essential services	The provision of the services must not cause harm to themselves, and the removal of other services in order to afford the services must not be seen as a greater harm	Patients must be seen to benefit from the services offered. There may be a range of perceptions about what outcomes are actually acceptable

Justice	The health-care services need to provide care to as many children as possible, within the resources available. All care cannot be provided to all	There are different communities that are affected by decisions around HCC, and communities should not be disadvantaged by the provision of HCC to individuals or to other communities	Patients should have access to care on the basis of need and likelihood of benefit

**Table 5:** Processes to be applied to the processes of resource allocation for health care.

Trust	The people affected by the process need to trust that the people implementing the health care will do their best to provide that care fairly and equitably

Transparency	The process of resource allocation should be open to comment, and the basis for decision making should be made public

Responsiveness	Should be mechanisms within the system to respond to changes in circumstances and established mechanisms to appeal against specific decisions

Consistency	Policies should be consistently applied regardless of the individuals involved

Inclusiveness	People who are affected by policies should be involved in the processes of developing those policies

Accountability	Clinicians whose patients are affected by the process need every opportunity to appeal against decisionsManagers and administrators need to have details of the resources available, the processes used to allocate those resources

The principles generally considered in biomedical ethics (51) include respect for autonomy, non-maleficence, beneficence, and justice. It may be useful to consider the implications of these principles when applied at different levels of the health service delivery (Table 4).

Kass (52) proposed that the following questions should be asked when evaluating potential public health interventions:

What are the goals of the proposed program (this includes both the overall goals and the short-term goals) and in particular who will benefit from this intervention?
How effective is the program at achieving the stated goals?
What are the known or the potential burdens of the program?
Can the burdens be minimized or are there alternative approaches?
Is the program administered fairly?
How can the benefits and burdens of the program be fairly balanced?

### What Are the Goals of the Proposed Program (This Includes Both the Overall Goals and the Short-Term Goals) and in Particular Who Will Benefit From This Intervention?

If one considers the possibility of pediatric intensive care in LICs, the overall goal would be a reduction in mortality, and the short-term goal would be more effective resuscitation of critically ill children presenting to the health-care services. The costs of pediatric intensive care may be particularly high (including the entire system of staffing, equipment, and support structures). At best, the introduction of pediatric intensive care services will benefit the small number of children who have already accessed health-care services and have access to the intensive care. By contrast, a wider definition of critical care (53), which includes the process of providing care to all children with life-threatening injury and/or illness, would have the potential to affect a much wider group of children and potentially at a much lower cost per child.

### How Effective Is the Program at Achieving the Stated Goals?

It is possible to extrapolate from international data to the possible impact of HCC, but outcomes may be very different in LIMCs. In the setting of cardiac surgery in Guatemala, the outcomes were initially worse than expected and took time and considerable investment to improve, despite the presence of surgeons with considerable experience and training in the USA (54). There is also evidence that the establishment of international quality intervention program may lead to substantial improvements in outcomes from high-cost procedures as demonstrated in pediatric cardiac surgery (55).

The provision of PICU is very unlikely to make a substantial impact on the mortality of children in LICs where there are numerous deaths and at best extremely limited resources available for PICU. In middle-income countries, there is much more likelihood of PICU making an impact on pediatric mortality. There is also the consideration that the training of personnel for services such as PICU may take many years, and thus it may be worth considering the introduction of PICU and investing in personnel training some time before it is likely to be implemented within a region.

There is certainly evidence that over time, the introduction of relatively HCC has improved outcomes in a number of settings (56, 57), particularly for oncological problems. There is also evidence that HCC in LICs can improve over a period of time (47).

A particular challenge for HCC is the requirement of most HCCs for ancillary disciplines including anesthesia (58, 59), pediatric surgery (59), medical imaging, etc.—each of these services is frequently required to implement HCC, and all are under pressure, individual HCC may be difficult to implement.

It is also essential to consider the place of the HCC within the overall context of the health services within that country. Particular in areas such as the care of critically ill children, the overall outcome will depend on the entire “pathway” of care and not simply on the intensive care component (13, 60).

One of the potential consequences of the differentials between private and public health care in LMICs is that both sectors may have a limited benefit from HCC. In the private sector, the limited number of people having access may limit the experience and expertise that can be achieved by therapeutic teams, while the majority of patients simply do not have access. In addition, the movement of expertise for HCC from the public to the private sector may further compromise the quality of care in the public sector.

### What Are the Known or the Potential Burdens of the Program?

The direct burden of HCC such as pediatric intensive care relates to the consumption of resources (financial and personnel) within the context of limited resources. An indirect burden of HCC such as pediatric intensive care is the large burden of illness and handicap that may affect both the children who survive PICU (61–63) and their parents (64). This may be extremely problematic in settings where long-term support and rehabilitation are poorly available. Thus, decision making within the HCC environment may include the need to limit interventions at levels that would not usually be considered appropriate in HICs.

A significant potential burden of HCC is the impact of that allocation of resources on other services. In situations where there are relatively rich resources, the implementation of HCC may have a very little impact on other services. Sadly, in LICs, the development of HCC is inevitably going to compromise care in some other area of the system, and it is essential that that impact is recognized, assessed, and included in the overall evaluation process.

A major factor in providing pediatric intensive care services within LMICs [or during disaster events (65)] is the process of developing reasonable strategies to allocate those resources (66), and it is important to consider how this problem will be addressed.

### Can the Burdens Be Minimized or Are There Alternative Approaches?

The burdens of HCC can be limited or minimized by strict definitions and decision making around what services can and should be provided and to whom. Ideally, the process of decision making should be transparent and public (66). One potential approach to the process of resource allocation, and particularly the allocation of resources to relatively expensive services such as intensive care is the accountability for reasonableness (A4R) process that was initially described in Canada (67).

As suggested above, it is also important to consider whether in fact less expensive care could be utilized to achieve the same or better outcomes. It has been well demonstrated in several settings that the use of “lower” technology such as nasal cPAP for severe bronchiolitis was associated with both lower costs and better outcomes (68).

### Is the Program Administered Fairly?

This question can be addressed at different phases. Initially, it may be important to carefully consider whether there are individuals or groups of people who are not receiving an equitable opportunity to access the HCC services. It may initially be possible to identify groups of people who for particular reasons (including geography) will not be able to access certain services as easily as others. There are also situations where it is clearly not possible to provide all care to all people (69, 70), and decisions have to made as to what therapy can and should be made available. Ideally, the processes for the allocation of the resources should be agreed to in a process involving the communities affected by those services (66). Generally, those processes will need to have characteristics such as accountability and transparency (see Table 5).

In the longer term, the question can only be fully answered if data are collected on who actually utilized the services and what outcomes were achieved. Carefully collected data provide the only real way in which the impact of a service can be assessed. Inherently, data capture should include not only information on those who accessed a service but also data on those who perhaps could or should have accessed those services.

### How Can the Benefits and Burdens of the Program Be Fairly Balanced?

The benefits of the program can be considered at the individual level. Guidelines for the provision of life-sustaining care in neonates (71), children (72), and adults (73) in HICs have highlighted the need to act in the best interests of the patient at all times. Clearly, there are situations where the application of HCC may actually prolong suffering, and there would be a substantial (although not universal) agreement that this should be avoided.

The training of health-care workers may be substantially affected by decisions made regarding HCC that will be offered in a particular region. The training required to offer HCC such as intensive care is potentially both time-consuming and expensive. The provision of training to health-care workers without a commitment to support the HCC is likely to lead to frustration and in many cases the migration of health-care workers to richer parts of the world. However, lack of opportunities to undergo training and implement therapies (such as surgery and anesthesia) may also lead to medical migration-associated health-care problems.

Decision making around the utilization of high-cost care may affect a number of people including the patient and the family, health-care workers, hospital and health-care managers, and politicians and people involved with the formulation and administration of policy at a provincial and/or national level. With the exponential growth of global health programs in areas such as North America, there are also an increasing number of people outside of the countries who have an interest and sometimes incentives (financial and otherwise) in changing the provision of HCC in LICs and LMICs.

A crucial aspect of balancing burdens and benefits relates to the standards that are expected and applied in the development of the HCC. Programs that have implemented oncology training and services in LIMCs have had to make decisions about the level of services that could safely be provided in those settings (74). The only way in which the impact of interventions can be monitored is by the collection of the appropriate data (both in the HCC service and in the services that are potentially affected) (45, 75). Bhutta (76) has highlighted the need (in research) to consider the realities and constraints in the countries where the research or clinical services being considered have to be implemented. In the setting of resource constraints, the actual care currently available and the risks that would be acceptable in clinical care may be very different to those where other resources are available.

The only way to address potential and actual ethical concerns in this setting is to make sure that information regarding who is involved in the process of providing the HCC must be clearly available to all the people involved in the process. In addition, the allocation of resources to data collection and interpretation is the only way in which it is possible to assess the impact of HCC and interventions. The allocation of limited resources to this monitoring may seem a relatively low priority, but in the longer term, it is the only way in which interventions can be assessed and thus provides a rational basis for ongoing decision making.

## Processes of Decision Making

In an ideal system, decisions regarding the implementation of health-care policies would be made: in a coherent way at every level of policy making (Table 4), by people with a clear understanding of both the costs and the achievable benefits of high-cost interventions (particularly relative to other lower cost interventions), and in a way that involves and takes into account the wishes and concerns of the people who are affected by those decisions and policies. Particular concerns in LICs are processes required to develop the expertise and organization structures (77) that are locally available. International groupings and organizations may be able to make a contribution in this regard.

It may be particularly challenging to address the relative contributions of managers in health-care systems to decision making. In the South African context, it has been pointed out that while there are substantially higher resources per capita in the private health-care system, much of those resources come from the contributions of the people who benefit from that system. If in an LIC, richer people are effectively paying for their own health services, is there a reasonable case to allow that? In that setting, it may be crucial to develop a detailed understanding of the proportion of the real costs that the public sector is bearing (including training costs, tax rebates, etc). It may also be critical to consider collaborative efforts to bring about mutually beneficial outcomes (78, 79), although these may be associated with risks.

Internationally, support for the collection of accurate data regarding the costs and outcomes of HCC for children in LMICs could provide a substantial evidence base for decision making in those areas. Importantly, the data collection should include the specifics of local contexts as far as possible.

A number of authors have addressed the processes that may be involved, with particular support for the use of the A4R approach (66, 80–83). There is strong evidence that this approach may be useful in addressing a range of health-care dilemmas (including access to HCC such as dialysis). However, there is a real need for ongoing research into decision-making processes in LIMCs (84, 85). Underlying that research is the need for a deeper understanding of the values that need to be incorporated into decision making (and which are not universally agreed to) (6, 7).

## Conclusion

Ethical decision making around the provision of HCC pediatric care in LMICs and LICs may be a complex process which requires a deep understanding of the context and the implications of any intervention. At the very least, the process needs to incorporate a realistic assessment of the context, the resources (both available and required), and the likely impact of the provision of that care. There is a very real risk that the implementation of high-cost pediatric care may have relatively poor outcomes, and even worse, the utilization of resources in this way may compromise other services with adverse outcomes for many children.

Ideally, all decision making should be transparent and should involve all the communities who are likely to be affected by those decisions. Frameworks from the public health environment may provide a useful addition to the standard bioethical approach.

## Author Contributions

The author confirms being the sole contributor of this work and approved it for publication.

## Conflict of Interest Statement

The author declares that the research was conducted in the absence of any commercial or financial relationships that could be construed as a potential conflict of interest.
